# The Spiritual Aspect of Pain: An Integrative Review

**DOI:** 10.1007/s10943-023-01890-9

**Published:** 2023-08-13

**Authors:** Rocío De-Diego-Cordero, Cristina Velasco-Domínguez, Alicia Aranda-Jerez, Juan Vega-Escaño

**Affiliations:** 1 Research Group PAIDI‐CTS 969 Innovation in HealthCare and Social Determinants of Health, Department of Nursing, Faculty of Nursing, Physiotherapy and Podiatry, University of Seville, 41009 Seville, Spain; 2 Faculty of Nursing, Physiotherapy and Podiatry, University of Seville, 41009 Seville, Spain; 3 Department of Nursing, Faculty of Nursing, Physiotherapy and Podiatry, University of Seville, C/ Avenzoar 6, 41009 Seville, Spain

**Keywords:** Pain management, Spirituality needs, Coping strategies, Spiritual interventions, Healthcare professionals, Nursing care

## Abstract

Pain is an unpleasant sensory and emotional experience that affects individuals in various ways involving biological, psychological, social, and spiritual aspects. There is currently no comprehensive treatment that effectively addresses all aspects of pain. This integrative review aimed to analyze the spiritual aspect of pain relief. Following the specified methodological criteria, a total of 20 articles were selected. There evidenced a lack of spiritual care provided by healthcare professionals, even though its correlation with pain and its potential benefits have been widely demonstrated in the literature. Fortunately, some patients put into practice existential and religious tools to self-control and cope with their pain, although not always with a successful response.

**Supplementary Information:**

The online version contains supplementary material available at 10.1007/s10943-023-01890-9.

## Introduction

Pain is an unpleasant sensory and emotional experience associated with actual or potential tissue damage. It has an individual and subjective component, and fulfills an adaptive function (Raja et al., 2020). Likewise, it is a complex and multidimensional phenomenon in which physiological, psychological, behavioral, spiritual, social, and cultural factors intervene (Rodríguez-Marín et al., 2021). Although studies evaluating a comprehensive approach to pain are scarce, there is unanimity in the fact that interdisciplinary and multidimensional treatment is beneficial (Goldman & Schafer, 2021).

Chronic pain is considered by the World Health Organization (WHO) a public health problem that affects around 25% of the world population, representing between 15 and 20% of all visits to the doctor (Martínez-Ruiz et al., 2021). Several studies report a higher prevalence of chronic pain among the elderly, among women, and among groups with a high risk of social exclusion (Baskozos et al., 2023).

Spirituality is a personal concept that often refers to an awareness of an inner self and a sense of connection to an unseen higher power, nature, or a purpose greater than oneself. Thus, spiritual care helps people find meaning and purpose in life, look to the future with hope, and keep rewarding personal and higher life force relationships (Potter et al., 2019b). Although the connection between healing and spirituality is still unknown, research has shown its positive effects on physical and mental health, quality of life, and health promotion and disease prevention behaviors (Potter et al., 2019b). In conclusion, dealing with spiritual issues in clinical practice can help patients recover their lives by providing meaning, hope, and healing (Balzer Riley, 2021).

Increasingly, patients seek treatments based on a spiritual approach (Goldman & Schafer, 2021). Some of the most widely supported modalities are traditional Chinese medicine with practices such as tai chi, acupuncture, and ayurveda (Walling et al., 2019). Bioenergetic therapies incorporate therapeutic touch and reiki among their methods, both techniques are based on energy manipulation (Vertino, 2019). Recent studies have shown the potential of mindfulness, as well as meditation and yoga, in changing the interpretation of pain. Other mind–body techniques such as deep breathing, guided visualization, and music therapy may be valuable in relieving pain (Walling et al., 2019). However, there are those who still doubt its benefits due to its lack of evidence in the available scientific literature (Vertino, 2019).

In the specific field of nursing care, previous studies have pointed out that nursing professionals must be trained to assess and adequately manage pain in accordance with the provisions of the American Nurse Association, since they are the ones who spend more time with patients (Potter et al., 2019a), implying this its multidimensional approach. In this regard, in another study focused on spiritual interventions carried out by nurses, it was shown that these interventions are effective in promoting mental health and well-being of patients, having an impact on physical results (de Diego-Cordero et al., 2022).

Until now, few studies have addressed the spiritual aspect of pain, and even fewer have addressed it from the perspective of holistic care.

For all these reasons, the aim of this review is to analyze the approach to pain from the spiritual dimension.

## Methods

An integrative review of existing literature was used to identify and analyze the research-based literature on the spiritual side of pain. Integrative review is suitable for investigating a phenomenon because it provides a global understanding of it, considering both quantitative and qualitative research through the empirical literature (Whittemore et al., 2014). To carry out the study, the authors identified five stages in conducting an integrative review: (a) Problem identification; (b) Literature search; (c) Data evaluation; (d) Analysis of data; and (e) Presentation (Souza et al., 2010).

### Research Question

This review was guided by the question “How is the spiritual aspect of pain addressed?”.

### Data Sources and Search Strategy

A comprehensive literature review was conducted using the electronic databases: PubMed, Scopus, and Web of Science (WOS). A 10-year period for the review period search was carried out, the period between 2012 and 2022, to retrieve the most up-to-date information.

The strategy to follow was elaborated from the MeSH health sciences descriptors: *chronic pain*, *spirituality, spiritual, religion, religions, faith, belief system, religious, religiousness and religiosity*; in combination of the Boolean operators AND and OR, whose resulting strategy.

**Fig. 1:**
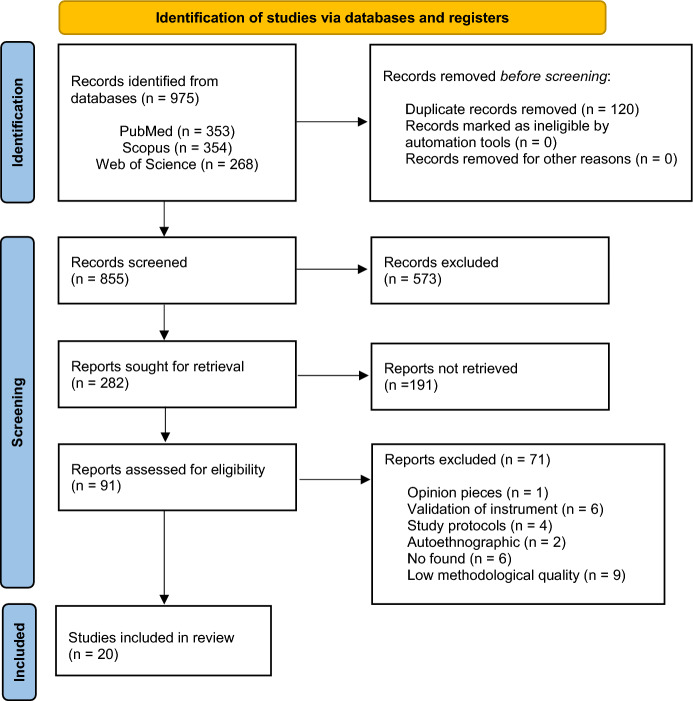
Flowchart for the selection of articles for the systematic review. *Note:* Page MJ, McKenzie JE, Bossuyt PM, Boutron I, Hoffmann TC, Mulrow CD, et al. The PRISMA 2020 statement: an updated guideline for reporting systematic reviews. BMJ 2021;372:n71. https://doi.org/10.1136/bmj.n71

All studies were then reviewed and evaluated to find those that met the established criteria for inclusion in this review. A flowchart was prepared according to the Preferred Reporting Items for Systematic reviews and Meta-Analyses for Protocols (PRISMA) (Page et al., 2021) (Fig. 1).

### Citation Management

A web-based bibliographic manager, Zotero 6.0.20, was used to collect and manage the citations included in the study.

### Eligibility Criteria

Articles were included if they: (a) whose study design included clinical trials, randomized controlled trials, meta-analyses, reviews, and systematic reviews; (b) were published between 2012 and 2022; (c) were published in peer-reviewed journals; (c) had original data and (d) whose language was English, Spanish, or German.

All documents whose type were books and book chapters, editorials and editorial materials, letters, notes, surveys, biographies, and meetings were excluded; as well as those that were study protocols, opinion articles, instrument validation, autobiographical articles or did not correspond to the objectives of the review.

The methodological quality was assessed using tools that ensure high-quality presentation of clinical trials (i.e., CONSORT), observational studies (i.e., STROBE), systematic reviews (i.e., PRISMA) and qualitative studies (i.e., SRQR guidelines) in order to determinate a sound methodology within the retrieved studies (see supplementary material). Studies scoring low on the appraisals were excluded.

### Procedure

Authors 2 and 3 conducted the searches individually and selected the studies that met the inclusion and exclusion requirements. Duplicate citations were initially manually discarded, titles and abstracts were then reviewed, and any disagreements were resolved by Author 1. The authors 2 and 3 reviewed the full text of selected articles to select those that included a spiritual aspect in pain treatment. Author 1 was responsible for resolving the disputes that arose in this process.

Article data extraction and analysis were performed by Author 4 and verified by Author 1. Any disagreements of these included articles were discussed until the team which reached a consensus, and the authors 2 and 3 independently prepared a results table. Finally, the data extracted included information about the authors, year, country, objective of the study, design, methodology and sample, instruments, results, and quality (Table 1).

**Table 1:** Summary of results

References, Country	Aim	Methods/sample	Instruments	Results
Andersen et al. (2019), Denmark	To explore how clinicians’ approach existential communication with their patients with chronic non-malignant pain, as well as its enablers and challenges	Systematic review of the literature		Physicians rarely meet the existential, spiritual, and religious needs of their nonmalignant chronic pain patients. Patient dissatisfaction with the physician's attention to these needs is related to increased pain and depression. The main facilitator was the individual disposition of the doctor to listen with openness and empathy to the existential concerns of his patients
Asadi-Piri et al. (2021), Iran	To investigate the relationship between pain self-efficacy and spirituality among older adults with chronic pain in Iran	Cross-sectional descriptive observational study *n* = 145	Spirituality Well-Being Scale-SWBS and Pain Self-Efficacy Questionnaire-PSE	The correlation between pain self-efficacy and the religious dimension was stronger than its correlation with the existential dimension; however, this difference was not statistically significant
Booker et al. (2020), USA	Expand understanding of personal experience managing osteoarthritis (OA) pain in older African Americans	Qualitative study *n* = 18 [Afro-Americans ≥ 50 age	Semi-structured interview (ad hoc script)	The concept of "bearing pain" is an expression and experience of living with chronic osteoarthritis pain that comprises 3 core actions: adapting to pain, sharing pain with others, and trusting God as a healer
Braun et al. (2022), Germany	To assess religiosity in patients with fibromyalgia syndrome. (FMS), its effect on pain and other symptoms, and on coping and FMS-related disability	Mixed study *n* = 102 [patients with fibromyalgia syndrome]	Medical records, ad hoc interviews, Aspects of Spirituality questionnaire. (ASP), Coping Strategies Questionnaire (CSQ), German Version of the Center of Epidemiological Studies General Depression Scale (CES-D), German version of the Pain Catastrophizing Scale (PCS), State-Trait Anxiety Inventory (STAI-G), Graded Chronic Pain Scale (GCPS), German Version of the Neuropathic Pain Scale Inventory (NPSI-D), and Fibromyalgia Impact Questionnaire (FIQ)	The degree of religiosity played a role in the choice of coping strategies but had no effect on health and mood. Depression and anxiety, coping "reinterpretation," catastrophizing, and pain intensity have a significant impact on disability due to FMS. Depending on the degree of disability, and in combination with other factors such as personal characteristics, stress management and life events; these five factors can increase or decrease resilience or vulnerability
Büssing et al. (2013), Germany	Identify unmet spiritual needs in patients with chronic pain conditions and cancer living in a secular society	Cross-sectional descriptive observational study *n* = 392	Spiritual Needs Questionnaire (SpNQ), Spirituality/Religiosity and Coping (SpREUK-15), Spiritual Well-being (FACIT-Sp), Brief Multidimensional Life Satisfaction Scale (BMLSS), Interpretation of Illness Questionnaire, Escape from Illness (Escape) and visual analog scale (VAS)	The religious and existential needs were of less relevance for the patients than those of inner peace and generation/active generativity. Safety (inner peace) needs include external enablers of intrinsically peaceful states (quiet places in the middle of nature) and internal enablers (finding inner peace, talking to others about fears and worries, turning to someone with a loving attitude) to alleviate the perceived “threat” of disease
Closs et al. (2013), England	To explore the relationships between religious identity and the experience and expression of chronic pain for five religious’ groups: Christians, Jews, Muslims, Hindus, and Sikhs	Systematic review of the literature		There is no available evidence on how the beliefs of the world's major religions impact the experience of chronic pain, or how they influence its expression. Furthermore, in most studies, religious identity was confined to Christianity, again with few consistent findings. Those studies that considered positive and negative religious and spiritual coping attempted to explore positive attitudes of love and care toward God (or a higher power) or negative attitudes such as anger and fear toward God, and the impact of these attitudes on experience from pain. The results of these studies were also weak
Feuille and Pargament (2013), USA	To examine mindfulness as an intervention for headache	Randomized controlled study *n* = 107	Ad hoc surveys, meditation/relaxation training scripts, ID-Migraine screener, Headache Impact Test (6-item version: HIT-6) and Toronto Mindfulness Scale (TMS)	Mindfulness training leads to a partial reduction in pain-related stress among people with migraine compared to simple relaxation techniques, providing modest support for their use in pain management. Furthermore, the spiritual content integrated into such training improves levels of mindfulness, although this does not correlate with better pain outcomes
Harris et al. (2018), USA	To examine veterans' spiritual distress as a predictor of two aspects of chronic pain, catastrophizing, and interference, testing a mediational model of depression	Cross-sectional descriptive observational study *n* = 436 [veterans with chronic pain]	Ad hoc survey, Patient Health Questionnaire (PHQ-8), Religious and Spiritual Struggles Scale (RSSS), Pain Catastrophizing Scale (PCS) and Pain Interference Scale (PIS)	Spiritual distress is a positive and significant predictor of both catastrophizing and pain interference. Furthermore, depression mediated, partially (in interference) or totally (in catastrophizing), the links between spiritual distress and grief outcomes. Therefore, the results suggest that spiritual distress may exacerbate chronic pain
Hasenfratz et al. (2021), Switzerland	To investigate the proportion and characteristics of chronic pain patients who want spiritual aspects to be integrated into their treatment	Cross-sectional descriptive observational study *n* = 209 [patients with chronic pain]	Ah hoc questionnaire, Hospital Anxiety and Depression Scale (HADS), Resilience Scale (RS-11), Spiritual and Religious Attitudes in Dealing with Illness (SpREUK), and 12-item Spiritual Well-Being Scale (FACIT-Sp-12)	Of the chronic pain patients who participated, 61.7% wanted spiritual aspects to be considered in their medical treatment. Those who indicated a desire to include spiritual aspects in the treatment of chronic pain were significantly younger, spiritual, had a higher academic education, had grown up primarily in Switzerland, and experienced higher levels of pain
Hatefi et al. (2019), Iran	To determine the relationship between religious coping (RC) and attachment to God with perceived pain in elderly people with chronic low back pain (CLBP) in Iran	Cross-sectional descriptive observational study *n* = 300 [older adults with chronic low back pain]	Ad hoc survey, Religion Coping Questionnaire, Attachment to God, Chronic Pain Acceptance and Visual Analog Scale for Pain	The higher the level of CR and attachment to God, the more likely it is that pain will be reduced by increasing its acceptance. Therefore, it is suggested to perform appropriate religious interventions to patients to reduce their pain state in order to improve their quality of life
Lee et al. (2014), USA	To analyze the efficacy and effectiveness of the range of active self-management complementary and integrative medicine therapies used for the treatment of chronic pain symptoms	Systematic review of the literature		Tai-chi practice seems safe with a rare rate of adverse events. All included studies demonstrated that tai chi was as effective, or more effective, than its control in relieving chronic pain symptoms, which is a slight recommendation in its favor Yoga is at least as effective as its control or more effective, and reporting of adverse events linked to poor training is relatively frequent but not serious
Najem et al. (2021), Lebanon	To identify if and how religious beliefs and attitudes can influence pain intensity, interference, beliefs and cognitions, emotions, and coping among patients with chronic musculoskeletal pain	Systematic review of the literature		Religiousness is associated with worse pain-related beliefs, cognitions, and emotions, but with better pain acceptance. However, contradictory results are found between religious beliefs and attitudes and the different domains of pain such as its intensity, disability and self-efficacy
Owens et al. (2016), USA	Study people with the ability to live well with persistent pain and obtain a description of their experiences in the context of living with pain	Mixed study *n* = 80 [patients with chronic pain]	Ad hoc interviews and Posttraumatic Growth Inventory (PTGI), 3-Dimensional Wisdom Scale (3D-WS), Gratitude Questionnaire (GQ-6), The Fetzer Forgiveness Scale (long form) and NEO Personality Inventory Revised (NEO-PI_R)	The positive approach to living well with pain enables more communicative pain reporting, provides positive role models for patients and clinicians, and contributes to a broader theoretical perspective on persistent pain
Perrin et al. (2021), Switzerland	To identify commonalities and differences in the perceptions of chronic pain patients (CPP) and healthcare professionals (HCP) on the integration of spiritual care in holistic pain management	Qualitative exploratory study *n* = 42 CPP and 34 HCP	Ad hoc interviews and questionnaire	CPPs emphasize the importance of HCPs recognizing their overall human wholeness, including the spiritual dimension, and would like to give spiritual concerns a greater importance in their therapy. HCPs express difficulties in addressing and discussing spiritual concerns and needs with chronic pain patients. Both parties want clarification of the context in which the spiritual dimension could be integrated into the treatment. They see the need for greater awareness and training of health professionals on how the spiritual dimension can be addressed in therapeutic interactions
Rettke et al. (2021), Switzerland	To examine the perspective of chronic pain patients on spiritual issues and their possible integration into the treatment process	Qualitative exploratory study *n* = 42 [patients with chronic pain]	Interviews and ad hoc questionnaire	Most of the participants are in favor of including the spiritual dimension in the treatment of chronic pain, although they emphasize that they should be the ones who have the opportunity to decide whether or not to integrate spiritual issues in their pain management process Being seen, recognized and treated as a whole person in all its dimensions is a central aspect of the therapeutic relationship
Seguin-Fowler et al. (2020), USA	To evaluate the feasibility of a yoga intervention, taken as a spiritual practice, designed to reduce pain and its results	Randomized controlled study *n* = 38 [women ≥ 60 age and with chronic pain]	surveys, Brief Pain Inventory (BPI), RAND 36-Item Short Form Survey, Senior Fitness Test, Brief Resilience Scale, and Community Healthy Activities Model Program for Seniors (CHAMPS) Activities Questionnaire for Older Adults	The findings support the feasibility and potential benefits of regular restorative yoga practice, as intervention participants experienced reductions in pain interference and improvements in energy and social functioning
Shropshire et al. (2019), USA	To assess differences in comfort and pain among older people in assisted living facilities who had chronic non-cancer pain and who used or did not use non-pharmacological interventions	Cross-sectional descriptive observational study *n* = 82 [patients with chronic pain]	Ad hoc questionnaires, Brief Pain Inventory (BPI) and General Comfort Questionnaire (GCQ)	Older people using non-pharmacological interventions and taking pain relievers had higher perceived comfort scores and lower pain scores than those using pain relievers alone. The most common non-pharmacological interventions were exercise, heat therapy, spiritual/religious activity, and listening to music
Snell et al. (2019), USA	To evaluate the prevalence of chronic non-malignant pain in a university occupational therapy clinic and provide recommendations to improve pain management in the clinic and hospital referral system	Mixed study *n* = 33	Ad hoc interviews and Graded Chronic Pain Scale 2.0 (GCPS)	The CREATION Health Model represents a holistic (addresses physiological, psychological, social, and spiritual aspects of care) approach to Biblically-based pain management. Three (choice of coping strategies, rest and development of interpersonal relationships) of the eight principles of this model have been selected to improve pain management within the clinic based on the specific needs of their patients
Vasigh et al. (2020), Iran	To determine the relationship between spiritual health and pain self-efficacy in a group of patients with chronic pain in Iran	Cross-sectional descriptive observational study *n* = 150 [patients with chronic pain]	Ad hoc questionnaire, Religion Well-Being Questionnaire and Chronic Pain Self-Efficacy Questionnaire (PSEQ)	Spiritual health is a predictor of pain acceptance, so religious patients were more likely to tolerate chronic pain. For this reason, it is suggested that religious interventions be carried out to reduce this type of pain
Yu et al. (2016), Dominican Republic	To investigate approaches to pain management and postoperative recovery prospects in patients with advanced arthritis who undergo total joint replacement (TJR) in the Dominican Republic	Qualitative study *n* = 20	Ad hoc interviews	The patients had strong religious beliefs that gave them strength to cope with chronic arthritis pain and prepare for acute pain after surgery. In the interviews they showed a lot of trust and hope that God and the doctors would heal their pain through surgery. Patients reported modest use of pain medications and limited knowledge of opioids, and many relied on nonpharmacologic therapies and family support to cope with their pain

### Quality Assessment of the Research Studies

Evaluation of the methodological quality of the selected investigations was carried out by using the tools contained in the Equator guidelines. Strobe (von Elm et al., 2014) for observational studies, Consort (Schulz et al., 2010) for clinical trials, and SRQR guidelines (O’Brien et al., 2014) for qualitative studies. This analysis was carried out by two authors (Author 2 and Author 3) and by a third author (Author 4) to resolve discrepancies. The appraisal scores of the studies were summarized in Table 1.

## Results

### Study Characteristics

The original search yielded 975 potentially relevant articles. After the first level of screening of articles for titles and abstracts, 402 articles remained for the second level of screening. After data characterization of the full-text articles of 91, a total of 20 studies were included in this review after removing unrelated studies.

Of the total, seven studies are cross-sectional descriptive observational studies, 4 bibliographic reviews, 4 qualitative studies, 3 mixed studies and 2 randomized controlled trials.

A total of 70% of the selected studies were published in the last 5 years. Most of the studies were carried out in the USA (40%), followed by Iran and Switzerland representing 15% of the sample, respectively, and Germany with 10%; while Denmark, England, Lebanon, and the Dominican Republic have a single record, which represents 5% for each of them.

In relation to the size of the sample, a great variety can be observed among the investigations carried out. For example, we found studies like the one by Harris et al. (2018) with 436 veterans with chronic pain, to others such as Booker et al. (2020) with 18 African Americans over 50 years of age, among other, which reflecting the diversity between the different studies.

The main pain disorders addressed by the studies include fibromyalgia (Braun et al., 2022), osteoarthritis (Booker et al., 2020; Yu et al., 2016) along with pain in the chronic back (Hatefi et al., 2019), musculoskeletal (Najem et al., 2021) and head (Feuille & Pargament, 2013). They also include entities that cause pain such as cancer (Büssing et al., 2013). Three of the reviewed papers focus on pain self-efficacy/self-control (Asadi-Piri et al., 2021; Lee et al., 2014; Vasigh et al., 2020) and 2 on interference with life or disability that causes pain (Braun et al., 2022; Harris et al., 2018), while one of them focuses on quality of life (Shropshire et al., 2019).

Regarding the study population, the majority (Asadi-Piri et al., 2021; Booker et al., 2020; Hatefi et al., 2019; Seguin-Fowler et al., 2020; Shropshire et al., 2019) are aimed at older people. Regarding the ethnographic origin, 3 of the investigations were carried out with the Iranian population (Asadi-Piri et al., 2021; Hatefi et al., 2019; Vasigh et al., 2020), 1 with African Americans (Booker et al., 2020) and another with Dominicans (Yu et al., 2016). Likewise, two of the publications dealt with the relationship between health professionals and patients (Andersen et al., 2019; Perrin et al., 2021). It is worth noting that one of the works focused specifically on women (Seguin-Fowler et al., 2020).

The large areas of research detected are, from more general to more specific, non-pharmacological interventions (Shropshire et al., 2019), movement therapies (Lee et al., 2014), yoga (Seguin-Fowler et al., 2020) and mindfulness (Feuille & Pargament, 2013).

Finally, the fields where the analyzes were implemented were university hospitals/health centers (Asadi-Piri et al., 2021; Braun et al., 2022; Hasenfratz et al., 2021; Hatefi et al., 2019; Vasigh et al., 2020; Yu et al., 2016), pain management clinics (Büssing et al., 2013; Harris et al., 2018; Rettke et al., 2021; Snell et al., 2019) and residences (Shropshire et al., 2019).

Regarding the instrument for data collection, 20% of the studies (qualitative) used only questionnaires and interviews not validated ad hoc, although in the other 20% of the studies (reviews of the literature) the use of any instrument. Within the investigations (quantitative and mixed), 60% used validated scales, the most repeated being: Visual Analog Scale (VAS), Spiritual Well-being Scale (FACIT-Sp), the Pain Catastrophizing Scale (PCS), Pain Self-Efficacy Questionnaire (PSEQ), Spiritual and Religious Attitudes in dealing with illness (SpREUK), Graded Chronic Pain Scale (GCPS) and Brief Pain Inventory (BPI).

### Main Issues

Related to the vision of patients and health professionals about spiritual care of pain, six studies answered to the question regarding both visions (Andersen et al., 2019; Booker et al., 2020; Hasenfratz et al., 2021; Owens et al., 2016; Perrin et al., 2021; Rettke et al., 2021; Yu et al., 2016). Four studies were qualitative. The other two are a systematic review of the literature (Andersen et al., 2019) and a mixed study (Owens et al., 2016). Most used a descriptive design to explain the different vision between chronic pain patients (CPP) but in healthcare professionals (HCP) there is not too much information about that.

In reference to the barriers of chronic pain self-management, the studies reviewed pointed out that physicians rarely meet the existential, spiritual, and religious needs of their nonmalignant CPP, their dissatisfaction with the physician's attention to these needs is related to increased pain and depression. In contrast, related to the facilitators of chronic pain self-management, the main facilitator was the individual disposition of the doctor to listen with openness and empathy to the existential concerns of his patients. It is a good point to recognize their overall human wholeness, including the spiritual dimension: being seen, recognized and treated as a whole person in all its dimensions is a central aspect of the therapeutic relationship. Those who indicated a desire to include spiritual aspects in the treatment of chronic pain (61.7% wanted spiritual aspects to be considered in their medical treatment) although they should be the ones who could decide whether or not to integrate spiritual issues in their pain management process. They know that they must bear the pain and they can conquer this experience with three actions: adapting to pain, sharing pain with others, and trusting God as a healer. The positive approach to living well with pain enables more communicative pain reporting, provides positive role models for patients and clinicians, and contributes to a broader theoretical perspective on persistent pain. In one qualitative study, the patients had strong religious beliefs that gave them strength to cope with chronic arthritis pain and prepare for acute pain after surgery. In the interviews they showed a lot of trust and hope that God and the doctors would heal their pain through surgery. Patients reported modest use of pain medications and limited knowledge of opioids, and many relied on nonpharmacologic therapies and family support to cope with Yu et al. (2016).

In relation with the strategies to work the spiritual dimension of pain, five studies answered to the question: strategies to work the spiritual dimension of pain (Feuille & Pargament, 2013; Lee et al., 2014; Seguin-Fowler et al., 2020; Shropshire et al., 2019; Snell et al., 2019). Yoga is at least as effective as its control or more effective, and reporting of adverse events linked to poor training is relatively frequent but not serious. The findings support the feasibility and potential benefits of regular restorative yoga practice, as intervention participants experienced reductions in pain interference and improvements in energy and social functioning. In addition, mindfulness training leads to a partial reduction in pain-related stress among people with migraine compared to simple relaxation techniques, providing modest support for their use in pain management. Furthermore, the spiritual content integrated into such training improves levels of mindfulness, although this does not correlate with better pain outcomes. And Tai chi practice seems safe with a rare adverse event rate. All included studies demonstrated that tai chi was as effective, or more so, than its control in relieving chronic pain symptoms, which is a slight recommendation in its favors.

Related to other non-pharmacological interventions, older people have used pain relievers had higher perceived comfort scores and lower pain scores than those using pain relievers alone. The most common non-pharmacological interventions were exercise, heat therapy, spiritual/religious activity, and listening to music. And the CREATION Health Model represents a holistic (addresses physiological, psychological, social, and spiritual aspects of care) approach to Biblically based pain management. Three (choice of coping strategies, rest and development of interpersonal relationships) of the eight principles of this model have been selected to improve pain management within the clinic based on the specific needs of their patients.

Finally, related to the relationship between hoping needs, resources spiritual and religious and pain of Patients in clinical practice, eight studies answered to the question: Booker et al. (2020), Braun et al. (2022), Büssing et al. (2013), Closs et al. (2013), Harris et al. (2018), Hatefi et al. (2019), Najem et al. (2021) and Vasigh et al. (2020).

The correlation between pain self-efficacy and religious was stronger than its correlation with the existential dimension; however, this difference was not statistically significant (Asadi-Piri et al., 2021). The degree of religiosity played a role in the choice of coping strategies but had no effect on health and mood (Braun et al., 2022). There is no available evidence on how the beliefs of the world's major religions impact the experience of chronic pain, or how they influence its expression. Furthermore, in most studies, religious identity was confined to Christianity, again with few consistent findings. Those studies that considered positive and negative religious and spiritual coping attempted to explore positive attitudes of love and care toward God (or a higher power) or negative attitudes such as anger and fear toward God, and the impact of these attitudes on experience from pain. The results of these studies were also weak (Closs et al., 2013). The higher the level of CR and attachment to God, the more likely it is that pain will be reduced by increasing its acceptance. Therefore, it is suggested to perform appropriate religious interventions to patients to reduce their pain state in order to improve their quality of life (Hatefi et al., 2019). Religiousness is associated with worse pain-related beliefs, cognitions, and emotions, but with better pain acceptance. However, contradictory results are found between religious beliefs and attitudes and the different domains of pain such as its intensity, disability and self-efficacy (Najem et al., 2021). Spiritual health is a predictor of pain acceptance, so more religious patients were more likely to tolerate chronic pain. For this reason, it is suggested that religious interventions be carried out to reduce this type of pain (Vasigh et al., 2020).

### Correlations Between Factors

In addition to the above, there are some correlations between some factors that should have taking in account.

First, the correlation between pain self-efficacy and other factors showed that depression and anxiety, coping “reinterpretation,” catastrophizing, and pain intensity have a significant impact on disability due to fibromyalgia syndrome FMS. Depending on the degree of disability, and in combination with other factors such as personal characteristics, stress management and life events; these five factors can increase or decrease resilience or vulnerability (Braun et al., 2022).

Second, the correlation between pain self-efficacy and inner peace, quiet people, and places. The religious and existential needs were of less relevance for the patients than those of inner peace and generation/active generativity. Safety (inner peace) needs include external enablers of intrinsically peaceful states (quiet places in the middle of nature) and internal enablers (finding inner peace, talking to others about fears and worries, turning to someone with a loving attitude) to alleviate the perceived “threat” of disease (Büssing et al., 2013).

Thirdly, related to spiritual distress and depression, spiritual distress is a positive and significant predictor of both catastrophizing and pain interference. Furthermore, depression mediated, partially (in interference) or totally (in catastrophizing), the links between spiritual distress and grief outcomes. Therefore, the results suggest that spiritual distress may exacerbate chronic pain (Harris et al., 2018).

## Discussion

The present systematic review aimed to analyze the approach to pain from the spiritual dimension. In general, the findings of this study suggest that to adequately treat pain, pharmacological measures should be complemented with therapies that include the fourth dimension of people: spirituality. According to the results obtained, the association between spiritual well-being and acceptance of pain translates directly into a reduction in its intensity, and those affected by various painful pathologies frequently resort to their religious and existential beliefs to deal with this condition in their lives. Other strategies with a spiritual component that are supported by evidence for pain management include yoga and mindfulness.

Considering patients' and professionals' perspectives on the spiritual approach to pain, there is evidence of patients' interest in integrating spiritual issues into their pain management and treatment (Rettke et al., 2021). In this regard, previous studies note that patients demand that their healthcare providers address their spiritual needs (Taylor et al., 2013), prefer to have spiritual aspects addressed in their chronic pain management (Hasenfratz et al., 2021) and want spiritual issues to be a potential topic to be explored during interactions with healthcare professionals (Perrin et al., 2021).

Regarding the coping of mourners, distraction, and mindfulness (meditation, music, nature or prayer) in addition to spirituality are shown as strategies for coping with chronic pain (Booker et al., 2020; Rettke et al., 2021) coupled with integrative and mind–body management therapies (meditation, yoga, gardening, art, music and nature) (Owens et al., 2016; Perrin et al., 2021; Yu et al., 2016).

With regard to the perception of health workers, a variety of results are evident: on the one hand, there is evidence that healthcare workers share the view that there are more important issues than spiritual care when talking to those affected (Perrin et al., 2021) and clinicians rarely show interest in existential needs as important, but rather as a secondary priority in care (Andersen et al., 2019); and on the other hand there are studies showing that healthcare workers care about religious characteristics and perceive this content as relevant in the hospital setting (Sohail et al., 2022).

In addition, the analysis of health professionals' perceptions of non-pharmacological measures for the management of chronic pain explains that the most common causes for the rejection of non-pharmacological measures are the lack of credibility that some patients and their relatives have for them, as they consider that only pharmacological measures are effective, given that their results are more immediate. In addition, participants recognize that this type of treatment does not have sufficient scientific evidence to support its efficacy, so that the rate of its use by professionals and institutions may increase (Garcia-Uribe & Castaño-Diez, 2022; Yu et al., 2016), with non-steroidal anti-inflammatory drugs (NSAIDs) being the most commonly used drugs since, for most patients, fear of side effects (including addiction to drugs such as tramadol) is a major concern leading to moderate use of analgesics (Álvarez et al., 2021; Yu et al., 2016).

In terms of facilitators and barriers experienced by experts in this spiritual approach to pain, the willingness and permission to free expression of patients are pointed out as facilitators and the predominance of the biomedical model, lack of time, lack of knowledge and skills (Andersen et al., 2019) as well as discomfort with spiritual-religious discussions (Perrin et al., 2021) as barriers.

Regarding the specific barrier of lack of training, both nursing and medical professionals state that the knowledge obtained during undergraduate studies is not sufficient to provide comprehensive management of patients with chronic pain (Garcia-Uribe & Castaño-Diez, 2022), pointing to chaplains as the specialists for the spiritual approach to pain (Carey et al., 2006; Gijsberts, 2022), although chronic pain patients frequently mentioned a predilection for certain professional groups (psychiatrists, anesthesiologists, nurses and physiotherapists) when discussing spiritual concerns (Perrin et al., 2021), highlighting that competences of team spirit, dealing with patients/communication and empowerment are more present in nurses compared to physicians and other health professionals (Sohail et al., 2022).

In terms of spiritual interventions that have been shown to be effective for pain management, the ACPA (2021) considers the use of drugs alongside other therapies as the cornerstone of pain management. Yoga, understood as a spiritual practice, stands out first (Deuel & Seeberger, 2020; Lee et al., 2014; Seguin-Fowler et al., 2020) or as an exercise modality (Palomino Alonso, 2015), supported by other studies that corroborate the advantages of using yoga to alleviate disability, pain and depression in people with chronic low back pain (American Chronic Pain Association, 2021) and to mitigate the motor symptoms of Parkinson's disease by producing improvements in balance, strength, posture and gait (Deuel & Seeberger, 2020) coupled with increased perceived social support in group sessions leading to greater acceptance and pain reduction (Lee et al., 2014; Seguin-Fowler et al., 2020). Another prominent intervention is mindfulness, which provides benefits with respect to migraine-related stress (Feuille & Pargament, 2013) as well as anxious, depressive and general psychological distress (Langer et al., 2017) and unpleasant experience of pain (American Chronic Pain Association, 2021).

Regarding spiritual/religious needs in pain patients, the needs for inner peace and generative relationship were the highest rated by patients with chronic pain and cancer diseases, even among those who considered themselves skeptical expressed specific religious needs such as praying (13%) or attending a religious service (13%) (Büssing et al., 2013). Regarding this association between religious affiliation and the experience or expression of pain, previous approaches have related conceptualizations of pain and suffering in the context of the major world religions (Judaism, Christianity, Islam, Hinduism and Buddhism) (Wachholtz et al., 2016).

In terms of coping strategies, a wide range of studies have examined the adaptive and maladaptive impact of spiritual and religious coping resources and their influence on pain, with relationships established between spiritual tendencies and serotonin receptor densities that regulate both mood and pain (Wachholtz et al., 2016), positive and negative pain acceptance (helplessness and lack of self-management) based on mental processing (Booker et al., 2020) or the use of passive coping strategies such as catastrophizing, ignorance and helplessness (Braun et al., 2022), with spiritual distress playing a telling role in catastrophizing and pain interference (Harris et al., 2018).

### Limitations

The current review has some limitations that should be taking in account. First, although the search strategy was thorough there may have been studies missed. Exclusion of some important sources such as grey literature could have resulted in the loss of potential important relevant results.

Second, the date limits used in this study have excluded older studies. Although this is a limitation, authors decided to include the most up-to-date evidence in this review, since guidelines, clinical practices and evidence change in a rapid pace.

## Conclusion

There are discrepancies between patients and health professionals as to the role that spiritual care should play in patient care, generating dissatisfaction during their encounters. Health professionals allege lack of time and training, finding differences with respect to other groups such as nurses who tend to be preferred for their more comprehensive treatment. Differences are also found in relation to the use of non-pharmacological measures that are widespread among mourners, but less recognized by health professionals.

Several spiritual therapies have been shown to be useful in the comprehensive approach to pain. Although yoga is not always practiced in these terms and can lead to injury, its practice has been shown to be effective in pain limitation and the socialization process. The repercussions of mindfulness are translated to a greater extent at the psychic level, which is a component also affected by the experience of pain. The use of non-pharmacological measures such as religion or spirituality has shown mixed but mainly beneficial results.

Individuals with pain, even non-believers, have existential needs, although one should not lose sight of the rest of a person's spheres. Among these, the approach of different religions in the construction of pain needs to be further explored, especially when considering that as societies become more secular, access to traditional religious resources weakens. For their part, spiritual coping strategies bring both benefits and detriments to the management of pain and other stressful situations in accordance with the established locus of control, which determines the coping styles that exist. Among the maladaptive coping mechanisms, catastrophism stands out, which some link to depression and others also to spiritual distress.

## Clinical Implications

These findings have important health implications. In the present review, we have identified that spirituality is an important resource for managing pain. This is especially important for health care managers, as they should be aware of the importance of this dimension and act to provide support for its approach in clinical practice, incorporating practices such as yoga or mindfulness as part of the therapeutic plan.

Finally, the shortage of randomized controlled trials in this review draws attention to the lack of strong evidence that spiritual interventions were able to treat pain.

## Recommendations for Future Research

The spiritual aspect of pain is insufficiently addressed in the current healthcare system, partly due to poor training of professionals, as well as misinformation about available treatment alternatives. Despite this, patients continue to demand attention to their spiritual needs.

More research studies are needed to incorporate established observational findings into clinical practice.

### Supplementary Information

Below is the link to the electronic supplementary material.

Supplementary file1 (DOCX 21 KB)

Supplementary file2 (DOCX 20 KB)

Supplementary file3 (DOCX 18 KB)

Supplementary file4 (DOCX 27 KB)
