# Supplementary material for: The Spiritual Aspect of Pain: An Integrative Review
Source: J Relig Health. 2023 Aug 13;63(1):159–84. doi: 10.1007/s10943-023-01890-9 (PMC10861647; doi:10.1007/s10943-023-01890-9)
Supplement: Supplementary file 1 — Supplementary file1 (DOCX 21 KB) [file 10943_2023_1890_MOESM1_ESM.docx]

**Table S1**: Quality assessment of the studies included in the systematic review (CONSORT Statement)

|  | Títle and abstract | | Introduction | | Trial desing | | Participans | | Interventions | Outcomes | | Sample size | | Secuencie generation | | Allocation  Concealment mechanism | implementation | Blinding | | Statistical methods | | Participant flow | | Recruitment | |
| --- | --- | --- | --- | --- | --- | --- | --- | --- | --- | --- | --- | --- | --- | --- | --- | --- | --- | --- | --- | --- | --- | --- | --- | --- | --- |
|  | 1 | | 2 | | 3 | | 4 | | 5 | 6 | | 7 | | 8 | | 9 | 10 | 11 | | 12 | | 13 | | 14 | |
|  | a | b | a | b | a | b | a | b |  | a | b | a | b | a | b |  |  | a | b | a | b | a | b | a | b |
| Feuille y Pargament, 2015. | 0.5 | 0 | 0.5 | 0.5 | 0 | 0.5 | 0.5 | 0.5 | 1 | 0.5 | 0 | 0.5 | 0.5 | 0.5 | 0 | 1 | 0 | 0.5 | 0.5 | 0.5 | 0 | 0.5 | 0.5 | 0.5 | 0.5 |
| Seguin-Fowler et al., 2020. | 0.5 | 0.5 | 0.5 | 0.5 | 0 | 0 | 0.5 | 0.5 | 1 | 0 | 0 | 0 | 0 | 0 | 0 | 1 | 0 | 0 | 0 | 0.5 | 0.5 | 0.5 | 0.5 | 0.5 | 0.5 |

(continued)

|  | Baseline data | Numbers analysed | Outcomes / estimation | | Ancillary analyses | Harms | Limitations | Generalisability | Interpretation | Registration | Protocol | Funding |  |
| --- | --- | --- | --- | --- | --- | --- | --- | --- | --- | --- | --- | --- | --- |
|  | 15 | 16 | 17 | | 18 | 19 | 20 | 21 | 22 | 23 | 24 | 25 |  |
|  |  |  | a | b |  |  |  |  |  |  |  |  |  |
| Feuille y Pargament, 2015. | 1 | 1 | 0.5 | 0.5 | 0 | 1 | 1 | 1 | 1 | 0 | 1 | 1 | 18.5 |
| Seguin-Fowler et al., 2020. | 1 | 1 | 0 | 0 | 0 | 0 | 1 | 0 | 1 | 0 | 1 | 1 | 16 |
