# Supplementary material for: The Spiritual Aspect of Pain: An Integrative Review
Source: J Relig Health. 2023 Aug 13;63(1):159–84. doi: 10.1007/s10943-023-01890-9 (PMC10861647; doi:10.1007/s10943-023-01890-9)
Supplement: Supplementary file 2 — Supplementary file2 (DOCX 20 KB) [file 10943_2023_1890_MOESM2_ESM.docx]

**Table S2**: Quality assessment of the studies included in the systematic review (PRISMA Statement)

|  | Title | Abstract | Rationale | Objectives | Eligibility criteria | Information sources | Search strategy | Slection process | Data collection process | Data items | | Study risk of bias assessment | Efect measures | Syntesis methods | | | | | | Reporting bias assessment | | Certainty assessment |
| --- | --- | --- | --- | --- | --- | --- | --- | --- | --- | --- | --- | --- | --- | --- | --- | --- | --- | --- | --- | --- | --- | --- |
|  | 1 | 2 | 3 | 4 | 5 | 6 | 7 | 8 | 9 | 10 | | 11 | 12 | 13 | | | | | | 14 | | 15 |
|  |  |  |  |  |  |  |  |  |  | a | b |  |  | a | b | c | d | e | f | |  |  |
| Andersen et al., 2019. | 1 | 1 | 1 | 1 | 1 | 1 | 1 | 1 | 1 | 0.5 | 0.5 | 1 | 0 | 0 | 0 | 0.16 | 0 | 0.16 | 0 | | 1 | 0 |
| Closs et al., 2013. | 1 | 0 | 1 | 1 | 1 | 1 | 0 | 1 | 1 | 0.5 | 0.5 | 1 | 0 | 0 | 0 | 0 | 0.16 | 0 | 0 | | 0 | 0 |
| Lee, Crawford y Schoomaker, 2014. | 0 | 1 | 1 | 1 | 1 | 1 | 1 | 1 | 0 | 0.5 | 0.5 | 1 | 1 | 0 | 0 | 0 | 0 | 0 | 0 | | 0 | 0 |
| Najem et al., 2021. | 1 | 1 | 1 | 1 | 1 | 1 | 1 | 1 | 1 | 0.5 | 0 | 1 | 1 | 0.16 | 0 | 0.16 | 0.16 | 0.16 | 0 | | 1 | 0 |

(continued)

|  | Study selection | | Study characteristics | Risk of Bias in studies | Results of individual studies | Results of syntheses | | | | Reporting biases | Certainty of evidence | Discussion | | | | Registration and protocol | | | Support | Competing interests | Availability of data, code and other materials |  |
| --- | --- | --- | --- | --- | --- | --- | --- | --- | --- | --- | --- | --- | --- | --- | --- | --- | --- | --- | --- | --- | --- | --- |
|  | 16 | | 17 | 18 | 19 | 20 | | | | 21 | 22 | 23 | | | | 24 | | | 25 | 26 | 27 |  |
|  | a | b |  |  |  | a | b | c | d |  |  | a | b | c | d | a | b | c |  |  |  |  |
| Andersen et al., 2019 | 0.5 | 0.5 | 1 | 0 | 0 | 0 | 0 | 0 | 0 | 0 | 0 | 0.25 | 0.25 | 0.25 | 0.25 | 0.3 | 0.3 | 0.3 | 1 | 1 | 1 | 19.22 |
| Closs et al., 2013. | 0.5 | 0.5 | 1 | 1 | 1 | 0.25 | 0 | 0 | 0 | 0 | 0 | 0.25 | 0.25 | 0.25 | 0.25 | 0 | 0 | 0 | 0 | 0 | 0 | 14.41 |
| Lee, Crawford y Schoomaker, 2014. | 0,5 | 0 | 1 | 1 | 1 | 0.25 | 0 | 0 | 0 | 0 | 0 | 0.25 | 0.25 | 0 | 0.25 | 0 | 0 | 0 | 1 | 1 | 1 | 17.5 |
| Najem et al., 2021. | 0,5 | 0,5 | 1 | 1 | 1 | 0.25 | 0.25 | 0.25 | 0.25 | 0 | 0 | 0.25 | 0.25 | 0 | 0.25 | 0.3 | 0.3 | 0 | 1 | 1 | 0 | 21.49 |
