# Supplementary material for: The Spiritual Aspect of Pain: An Integrative Review
Source: J Relig Health. 2023 Aug 13;63(1):159–84. doi: 10.1007/s10943-023-01890-9 (PMC10861647; doi:10.1007/s10943-023-01890-9)
Supplement: Supplementary file 3 — Supplementary file3 (DOCX 18 KB) [file 10943_2023_1890_MOESM3_ESM.docx]

**Table S3**: Quality assessment of the studies included in the systematic review (SRQR Statement)

|  | Title | Abstract | Problem formulation | Purpose or research question | Qualitative approach and research paradigm | Researcher characteristics and reflexivity | Context | Sampling strategy | Ethical issues pertainining to human subjects | Data collection methods | Data collection instruments and technologies | Units of study | Data processing | Data analysis | Tecniques to enhance trstworthiness | Synthesis and interpretation | Links to empirical data | Discussion | Limitations | Conflict of interest | Funfing |  |
| --- | --- | --- | --- | --- | --- | --- | --- | --- | --- | --- | --- | --- | --- | --- | --- | --- | --- | --- | --- | --- | --- | --- |
|  | 1 | 2 | 3 | 4 | 5 | 6 | 7 | 8 | 9 | 10 | 11 | 12 | 13 | 14 | 15 | 16 | 17 | 18 | 19 | 20 | 21 |  |
| Booker, S. Q., 2020. | 0 | 0 | 0 | 1 | 0 | 0 | 1 | 1 | 0 | 0 | 1 | 1 | 1 | 1 | 0 | 1 | 1 | 0 | 1 | 1 | 1 | 12 |
| Braun et al., 2022. | 0 | 0 | 1 | 0 | 0 | 0 | 1 | 1 | 1 | 1 | 1 | 1 | 0 | 0 | 0 | 1 | 0 | 1 | 0 | 1 | 1 | 11 |
| Owens et al., 2016. | 1 | 1 | 0 | 0 | 1 | 1 | 1 | 1 | 1 | 1 | 1 | 0 | 1 | 1 | 1 | 1 | 0 | 0 | 1 | 1 | 1 | 16 |
| Perrin et al., 2021. | 1 | 1 | 1 | 1 | 0 | 1 | 0 | 0 | 1 | 1 | 1 | 1 | 1 | 1 | 1 | 1 | 1 | 0 | 1 | 1 | 1 | 17 |
| Rettke et al., 2021. | 0 | 1 | 1 | 1 | 1 | 1 | 1 | 1 | 1 | 1 | 1 | 1 | 0 | 1 | 1 | 1 | 1 | 1 | 1 | 1 | 1 | 19 |
| Snell et al., 2019. | 0 | 0 | 1 | 1 | 0 | 1 | 1 | 1 | 0 | 1 | 1 | 1 | 0 | 1 | 0 | 0 | 0 | 0 | 1 | 0 | 1 | 11 |
| Yu et al., 2016 | 1 | 0 | 0 | 0 | 0 | 1 | 1 | 1 | 1 | 1 | 0 | 1 | 0 | 0 | 1 | 1 | 1 | 1 | 1 | 0 | 1 | 13 |
