# Supplementary material for: The Spiritual Aspect of Pain: An Integrative Review
Source: J Relig Health. 2023 Aug 13;63(1):159–84. doi: 10.1007/s10943-023-01890-9 (PMC10861647; doi:10.1007/s10943-023-01890-9)
Supplement: Supplementary file 4 — Supplementary file4 (DOCX 27 KB) [file 10943_2023_1890_MOESM4_ESM.docx]

**Table S4**: Quality assessment of the studies included in the systematic review (STROBE Statement)

|  | Title and abstract | | Background / rationale | Objetives | Study desing | Setting | Participants | | Variables | Data sources / measurement | Bias | Study size | Quantitative variables | Statistical methods | | | | |
| --- | --- | --- | --- | --- | --- | --- | --- | --- | --- | --- | --- | --- | --- | --- | --- | --- | --- | --- |
|  | 1 | | 2 | 3 | 4 | 5 | 6 | | 7 | 8 | 9 | 10 | 11 | 12 | | | | |
|  | a | b |  |  |  |  | a | b |  |  |  |  |  | a | b | c | d | e |
| Asadi-Piri et al., 2021 | 0 | 0.5 | 1 | 0 | 1 | 1 | 0.5 | 0.5 | 1 | 1 | 1 | 1 | 0 | 0 | 0 | 0.2 | 0 | 0 |
| Braun et al., 2022. | 0 | 0.5 | 1 | 1 | 1 | 1 | 1 | np | 0 | 1 | 0 | 0 | 0 | 0.2 | 0.2 | 0 | 0 | 0.2 |
| Büssing et al., 2013. | 0.5 | 0.5 | 1 | 1 | 1 | 1 | 1 | np | 1 | 1 | 0 | 0 | 1 | 0 | 0 | 0 | 0 | 0.2 |
| Harris et al., 2018. | 0 | 0.5 | 1 | 1 | 1 | 1 | 1 | np | 0 | 1 | 0 | 1 | 1 | 0.2 | 0.2 | 0 | 0 | 0 |
| Hasenfratz et al., 2021 | 0.5 | 0.5 | 1 | 0 | 1 | 1 | 0.5 | 0.5 | 1 | 1 | 1 | 1 | 1 | 0 | 0 | 0.2 | 0 | 0 |
| Hatefi, Tarjoman y Borji, 2019 | 0.5 | 0.5 | 1 | 0 | 1 | 1 | 0 | np | 0 | 0 | 0 | 1 | 1 | 0.2 | 0.2 | 0.2 | 0.2 | 0.2 |
| Owens et al., 2016 | 0.5 | 0.5 | 0 | 0 | 0 | 0 | 0.5 | 0.5 | 0 | 1 | 1 | 1 | 1 | 0 | 0 | 0 | 0 |  |
| Shropshire et al., 2019. | 0.5 | 0.5 | 1 | 1 | 1 | 1 | 1 | np | 1 | 1 | 1 | 0 | 1 | 0.2 | 0.2 | 0 | 0 | 0.2 |
| Snell et al., 2019. | 0 | 0.5 | 1 | 1 | 0 | 1 | 1 | np | 0 | 1 | 0 | 1 | 0 | 0 | 0 | 0 | 0 | 0 |
| Vasigh, Tarjoman y Borji, 2020. | 0.5 | 0.5 | 1 | 1 | 1 | 1 | 1 | np | 0 | 1 | 0 | 1 | 0 | 0 | 0.2 | 0.2 | 0.2 | 0 |

(continued)

|  | Participants | | | Descriptive data | | | Outcomes data | Main results | | | Other analyses | Key results | Limitations | Interpretation | Generalisability | Funding |  |
| --- | --- | --- | --- | --- | --- | --- | --- | --- | --- | --- | --- | --- | --- | --- | --- | --- | --- |
|  | 13 | | | 14 | | | 15 | 16 | | | 17 | 18 | 19 | 20 | 21 | 22 |  |
|  | a | b | c | a | b | c |  | a | b | c |  |  |  |  |  |  |  |
| Asadi-Piri et al., 2021 | 0 | 0 | 0 | 0.5 | 0 | 0 | 1 | 0 | 0.5 | np | 0 | 1 | 0 | 1 | 0 | 1 | 13.7 |
| Braun et al., 2022. | 0.3 | 0.3 | 0.3 | 0.3 | 0 | 0 | 0 | 0.5 | 0.5 | np | 1 | 1 | 0 | 1 | 0 | 1 | 13.4 |
| Büssing et al., 2013. | 0 | 0 | 0 | 0.5 | 0.5 | np | 1 | 0.5 | 0.5 | np | 1 | 1 | 1 | 1 | 0 | 0 | 16.2 |
| Harris et al., 2018. | 0.3 | 0.3 | 0 | 0.5 | 0 | np | 1 | 0.5 | 0.5 | np | 0 | 1 | 1 | 1 | 0 | 1 | 16 |
| Hasenfratz et al., 2021 | 0 | 0 | 0 | 0,5 | 0 | np | 0 | 0 | 0 | 0 | 0 | 1 | 1 | 1 | 1 | 1 | 15.52 |
| Hatefi, Tarjoman y Borji, 2019 | 0.3 | 0.3 | 0.3 | 0 | 0 | 0 | 0 | 0 | 0 | 0 | 0 | 0 | 0 | 1 | 1 | 1 | 11 |
| Owens et al., 2016 | 0 | 0 | 0 | 0 | 0 | np | 1 | 0 | 0 | 0 | 1 | 1 | 1 | 0 | 0 | 1 | 11 |
| Shropshire et al., 2019. | 0 | 0 | 0 | 0.3 | 0 | 0 | 0 | 0.5 | 0 | np | 1 | 1 | 1 | 1 | 1 | 0 | 16.4 |
| Snell et al., 2019. | 0.3 | 0 | 0 | 0.5 | 0 | np | 1 | 0 | 0 | np | 0 | 0 | 1 | 1 | 0 | 1 | 11.3 |
| Vasigh, Tarjoman y Borji, 2020. | 0 | 0 | 0 | 0.5 | 0 | np | 0 | 0 | 0 | 0 | 0 | 0 | 1 | 1 | 1 | 0 | 11.06 |
